# Gestational weight gain charts for Latin American adolescents

**DOI:** 10.1371/journal.pone.0292070

**Published:** 2023-11-01

**Authors:** Sandra Lucía Restrepo-Mesa, María Victoria Benjumea Rincón, Alejandro Estrada Restrepo, Thais Rangel Bousquet Carrilho, Gilberto Kac, Josué Santiago Cano Pulgarín, Keren Cano-Pulgarín, Cecilia Severi, Odalis Sinisterra, María del Carmen Zimmer Sarmiento, Maria Isabel López Ocampos, Marcela Araya Bannout, Gabriela Chico-Barba, Nelida Pinto Arteaga, Carlos Grandi, Eduardo Atalah Samur, Cristian David Santa Escobar

**Affiliations:** 1 Research Group on Food and Human Nutrition, School of Nutrition and Dietetics, University of Antioquia, Medellín, Colombia; 2 University of Antioquia, Medellín, Colombia; 3 Research Group on Demographics and Health, School of Nutrition and Dietetics, University of Antioquia, Medellín, Colombia; 4 Nutritional Epidemiology Observatory, Josué de Castro Nutrition Institute, Federal University of Rio de Janeiro, Rio de Janeiro, Brazil; 5 Faculty of Medicine, Preventive Medicine Department, University of Republic, Montevideo, Uruguay; 6 Panama Ministry of Health, Panama City, Panama; 7 Faculty of Health Sciences, National University of Salta (UNSa), Salta, Argentina; 8 Health Promotion Section, Loma Pyta Maternal and Child Hospital, Public Health and Social Welfare Ministry, Asunción, Paraguay; 9 Faculty of Medicine, Women and Newborn Health Promotion Department, Chile University, Santiago, Chile; 10 Nutrition and Bioprogramming Coordination, National Institute of Perinatology, Ciudad de México, México; 11 Maternal Perinatal institute, Lima, Perú; 12 Pediatric Research Department, Argentine Society of Pediatrics, Buenos Aires, Argentina; 13 Faculty of Medicine, University of Chile, Santiago, Chile; Universidade Federal do Maranhão: Universidade Federal do Maranhao, BRAZIL

## Abstract

Monitoring gestational weight gain (GWG) throughout pregnancy among adolescents is important for detecting individuals at risk and timely intervention. However, there are no specific tools or guidelines for GWG monitoring of this group. We aimed to construct GWG charts for pregnant adolescents (10–19 years old) according to pre-pregnancy body mass index (BMI) using a pooled dataset from nine Latin American countries. Datasets from Argentina, Brazil, Chile, Colombia, Mexico, Panama, Paraguay, Peru, and Uruguay collected between 2003 and 2021 were combined after data cleaning and harmonization. Adolescents free of diseases that could affect GWG and who gave birth to newborns weighing between 2,500–4,000 g and free of congenital malformations were included. Multiple imputation techniques were applied to increase the sample size available for underweight and obesity categories. Generalized Additive Models for Location, Scale, and Shape were used to construct the charts of GWG according to gestational age. Internal and external validation procedures were performed to ensure that models were not over-adjusted to the data. The cohort included 6,414 individuals and 29,414 measurements to construct the charts and 1,684 individuals and 8,879 measurements for external validation. The medians (and interquartile ranges) for GWG at 40 weeks according to pre-pregnancy BMI were: underweight, 14.9 (11.9–18.6); normal weight, 14.0 (10.6–17.7); overweight, 11.6 (7.7–15.6); obesity, 10.6 kg (6.7–14.3). Internal and external validation showed that the percentages above/below selected percentiles were close to those expected, except for underweight adolescents. These charts describe the GWG throughout pregnancy among Latin American adolescents and represent a significant contribution to the prenatal care of this group. GWG cut-offs based on values associated with lower risks of unfavorable outcomes for the mother-child binomial should be determined before implementing the charts in clinical practice.

## Introduction

Gestational weight gain (GWG) is an important indicator of maternal nutritional status. Weight gain in this period is fundamental to promoting the health of the mother-child binomial, storing the necessary fat for milk production and adequate fetal growth and development, allowing for optimal weight and body composition in the newborn [1,2].

Deviations in GWG are associated with adverse outcomes for the mother-child binomial. Excessive GWG increases the probability of developing hypertensive disorders of pregnancy, gestational diabetes, cesarean delivery, postpartum weight retention and, in the newborn, it may increase the risk of birth of large-for-gestational-age infants, macrosomia, and, in the long term, childhood obesity [3–7]. In contrast, insufficient GWG may result in an inadequate reservoir to support lactation in the mother and the newborn, it may increase the probability of intrauterine growth restriction, small-for-gestational-age, low birth weight, and preterm birth [2,4,8].

Monitoring GWG throughout pregnancy is important for detecting at-risk individuals and timely intervention, especially among adolescents who face pregnancy under biological and psychological immaturity conditions, increasing the risk of unfavorable outcomes for mother-child binomial health [9]. According to the World Bank [10], by 2021, Latin America and the Caribbean had the second-highest adolescent birth rate globally (53.2 births per 1,000 adolescents), surpassed only by Sub-Saharan Africa (101 births per 1,000 adolescents). Teenage pregnancy rates in 2020 exceeded 60 births per 1,000 women in countries such as Venezuela, Panama, Colombia, Argentina, and Paraguay. In Mexico, Uruguay, Brazil, and Peru, pregnancy rates between adolescents were between 50 and 60 births per 1,000 women, while in Chile, this rate is approximately 40 births [10].

The World Health Organization (WHO) recommends GWG as an indicator to be monitored during pregnancy [11]. However, despite the magnitude of adolescent pregnancy and its implications for the health of the mother and the child, there are no proposals to assess GWG in this group nor specific guidelines or differentiated care for adolescents. This situation has led countries to use GWG charts developed for adult women [12], which have not considered the specific characteristics of adolescents in their design and have also been constructed with different methodologies, anthropometric indicators, and cut-off points for estimating GWG, which limits comparability between countries. Thus, the objective of this study was to construct GWG charts for pregnant adolescents from Latin America, according to pre-pregnancy body mass index (BMI), to contribute to the nutritional monitoring of this group during this phase of life.

## Methods

### Study design and sample

An observational, retrospective, longitudinal study was conducted based on secondary data from research projects, information systems, maternal clinical records, and prenatal care institutions in Argentina, Brazil, Chile, Colombia, Mexico, Panama, Paraguay, Peru, and Uruguay. The initial database comprised data from 33,446 women with approximately 150,000 weight measurements. After the data harmonization process by Standardized Site Differences (SSD) technique, 6,414 pregnant adolescents and 34,943 weight records were included in the study (S1 Fig).

We included women between 10 and 19 years old; with at least one weight measurement (kg) in the second and third trimesters of pregnancy, with their respective gestational age (weeks); without hypertension, preeclampsia, diabetes mellitus or gestational diabetes, tuberculosis, or cardiovascular diseases; and who gave birth to a term newborn (37 to 42 weeks), with birth weight between 2,500 and 4,000 g. In addition, records with a biologically implausible weight gain trajectory (very high weight gains or losses between controls), total GWG ≥ 30 kg, height-for-age z score (HAZ) <-2 according to the WHO charts [13] (childhood stunting), and no data on pre-pregnancy weight or BMI were excluded. The methodological details regarding the database constitution, cleaning, and data harmonization are described in detail elsewhere [14].

### Main variables

Maternal age in years was estimated as the difference between the date of birth of the pregnant adolescents and the date of conception. The date of conception was estimated from the gestational ages available in the databases using the ’ob Wheel’ algorithm (https://obwheel.quartertone.net/). Gestational age was already calculated in the combined datasets, and it was not possible to know how it was obtained (whether from ultrasonography or based on the last menstrual period date). Pre-pregnancy BMI (kg/m^2^) was classified according to the WHO criteria for adolescents as underweight: <-2 SD; normal weight: ≥ -2 SD to ≤ +1 SD; overweight: > +1 SD to ≤ +2SD; and obesity: > +2 SD [13]. GWG (kg) was calculated as the difference between the weight in each visit and pre-pregnancy weight, self-reported or abstracted from clinical records. The heterogeneity of the GWG was verified within each pre-pregnancy BMI category, and the data was homogeneous across the nine participating countries [14]. Birth weight and length were available in the datasets from each country. The homogeneity of the GWG and height-for-age z scores and similar maternal age distributions across the included countries reinforce the possibility of combining the data into a single dataset to construct GWG graphs for adolescents aged 10–19 years.

### Statistical analysis

Medians and interquartile ranges (IQR) were calculated for height (cm), pre-pregnancy weight (kg), maternal age (years), gestational age at birth (weeks), birth weight (g), birth length (cm), and GWG (kg) according to the pre-pregnancy BMI category.

A proportion of records of GWG that varied from 10.4% (underweight) to 12.8% (overweight) did not present values between the fifth and thirteen weeks of pregnancy (first trimester), which generated an overdispersion (high variance) in the data. Thus, the adjustments of the models to build the GWG charts were complex. The overdispersion of the data was especially present among adolescents with pre-pregnancy under and overweight. Thus, for those women who did not have a weight measurement in the first trimester, it was assumed that the lack of a weight measurement in this gestation period was a real observation that could be imputed. These new observations were assumed as missing not at random (MNAR), and a data imputation approach was considered for GWG in the first trimester. For each pregnant adolescent with missing data, a random week was established between the fifth and thirteenth week to expand the data matrix [14].

Subsequently, the Linear Regression Bootstrap imputation method [15] was applied, which extracted a bootstrap sample from the complete part of the data to estimate the coefficients of the model by minimal squares iteratively and to each missing data found the case that most closely agreed with the prediction. The method considered the longitudinal nature of the data. The adolescent gestational age at each prenatal visit (in weeks) and the number of prenatal visits during pregnancy were included as covariates in the imputation model. For these multiple imputations, Rubin’s rules were used to combine the results, and 10 rounds of imputations were performed (m = 5) [16].

The selection of the best imputation method to control for overdispersion in the first trimester (in individuals with pre-pregnancy BMI under and overweight) took into account the Generalized Akaike Information Criterion (GAIC) [17], the Akaike Information Criterion (AIC) and global deviation methods [18]. For all BMI categories, the diagnostic of the model comprised the evaluation of normality using QQ plots [19] and Mardia’s test [20].

To construct the charts of GWG according to gestational age for each pre-pregnancy BMI, generalized additive models of location, scale, and shape (GAMLSS) were used [18]. The models were fitted using Box-Cox Power Exponential (BCPE) and Box-Cox t-student (BCT) distributions. Because these distributions are defined for positive real numbers, a 20-kg constant was added to the GWG measurements to ensure that all records were positive. Using these models, we obtained the equations to calculate GWG z scores considering the four parameters modeled in GAMLSS. Eq 1 presents the equation for calculating the z scores and an example of its use.

**Eq1.** Equation for calculation of pre-pregnancy body mass index-specific gestational weight gain z-scores, based on a Box-Cox t or Box-Cox Power Exponential model, where Y is the weight gain at a given gestational age, ν is lambda, μ is mu, and σ is sigma. The random variable Z is assumed to follow a t-distribution with degrees of freedom, τ > 0, treated as a continuous parameter. Parameters of BCT or BCPE model for each pre-pregnancy BMI group are given for rounded gestational ages. To fit the GAMLSS models, the parameters were calculated based on weight gain + 20 kg, so 20 kg must be added to the weight gain when using this equation.

$$Z=\left\{ \begin{array}{l} \frac{1}{\sigma \nu }[{\left(\frac{Y+20}{\mu }\right)}^{\nu }-1]\;if\;\nu \neq 0\\ \frac{1}{\sigma }ln(\frac{Y+20}{\mu })\;if\;\nu =0 \end{array}\right.$$

Consider an adolescent with a normal pre-pregnancy BMI, with GWG of 5kg at week 15. According to S3 Table in S1 File, the following parameters are found μ = 21.54 σ = 0.12 ν = 0.08 τ = 4.24, as ν≠0. Thus:

$$Z=\frac{1}{\left(0.12)(0.08\right)}\left[{\left(\frac{5+20}{21.54}\right)}^{0.08}-1\right]=1.23$$

This z-score of 1.23 is equivalent to the percentile 89.06%.

The biological plausibility of the predicted GWG values for each model was evaluated, emphasizing the presence of negative GWG for each category of pre-pregnancy BMI and the magnitude of weight loss throughout pregnancy (between the first and third trimesters). The smoothing of the graphs in the first trimester and the percentile amplitude of GWG were also considered in the decision for the final models. Additionally, the internal validation of the model was carried out by calculating the percentage of measurements above/below selected percentiles. For example, if the model has good internal validation, 10% of the observations are expected to be below the 10^th^ percentile and 10% above the 90^th^ percentile. For the external validation of the model, the machine learning cross-validation methodology was used [21]. In this approach, the datasets for each pre-pregnancy BMI category were divided into 70% for the training of the GAMLSS model and 30% for the external validation. In the 30% dataset, the percentage of observations above/below selected percentiles was also calculated. The internal and external validation results were considered satisfactory if the difference between the expected and the obtained percentages did not exceed 5%. All statistical analyses were carried out in R version 4.0.3.

### Ethics

This study was approved by the Bioethics Committee of the Faculty of Dentistry of the Universidad de Antioquia, act number 12 of October 18, 2019. Only de-identified data was used in the analyses, which were performed by authorized investigators. The principal investigator of the University of Antioquia signed a confidentiality and custody agreement for the data with the investigator or institutional representative of each country.

## Results

A total of 6,414 pregnant adolescents aged 10 to 19 and their neonates were included. Most adolescents were classified with pre-pregnancy normal weight (71.8%), and only 1.5% were underweight. The median birth weight increased progressively as the pre-pregnancy BMI category increased (Table 1). In the first trimester of pregnancy, fewer measurements were available, especially for underweight and obesity categories, between 5 and 13 weeks (S1 Table in S1 File).

**Table 1:** Characteristics of Latin American pregnant adolescents aged 10 to 19 years and their newborns (n = 6,414 women).

Quantitative variables	Pre-pregnancy BMI category (SD from the WHO charts)				
	Total (n = 6,414)	Underweight (< - 2 SD) (n = 94)	Normal weight (≥ -2 SD and ≤ +1 SD) (n = 4,603)	Overweight (> +1 SD and ≤ +2SD) (n = 1,374)	Obesity (> + 2 SD) (n = 343)
	Median (P25—P75)	Median (P25—P75)	Median (P25—P75)	Median (P25—P75)	Median (P25—P75)
Maternal age (years)	17 (16–18)	18 (16–18)	17 (16–18)	17 (16–18)	17 (16–19)
Pre-pregnancy weight (kg)	54 (49–60)	40 (37.2–42)	52 (48–55)	62 (58.5–67)	75 (70–82)
Maternal height (cm)	157 (152–161)	160 (157–162)	157 (153–161)	155 (151–159)	156 (152–160)
Gestational age at birth (weeks)	37 (35–39)	37 (33–38)	37 (35–39)	37 (34–39)	37 (35–39)
Birth weight (g)	3,212 (2,980–3,470)	3,070 (2,906.3–3,438.7)	3,200 (2,960–3,450)	3,280 (3,024–3,520)	3,300 (3,406.5–3,519)
Birth length (cm)	49.5 (48–51)	49.8 (48–50)	49.0 (48–50.6)	50.0 (48.5–51)	50.0 (49–51)

Notes: n refers to the number of adolescents. BMI: Body mass index; IQR: Interquartile range; SD: Standard deviation; WHO: World Health Organization.

**Fig 1:**
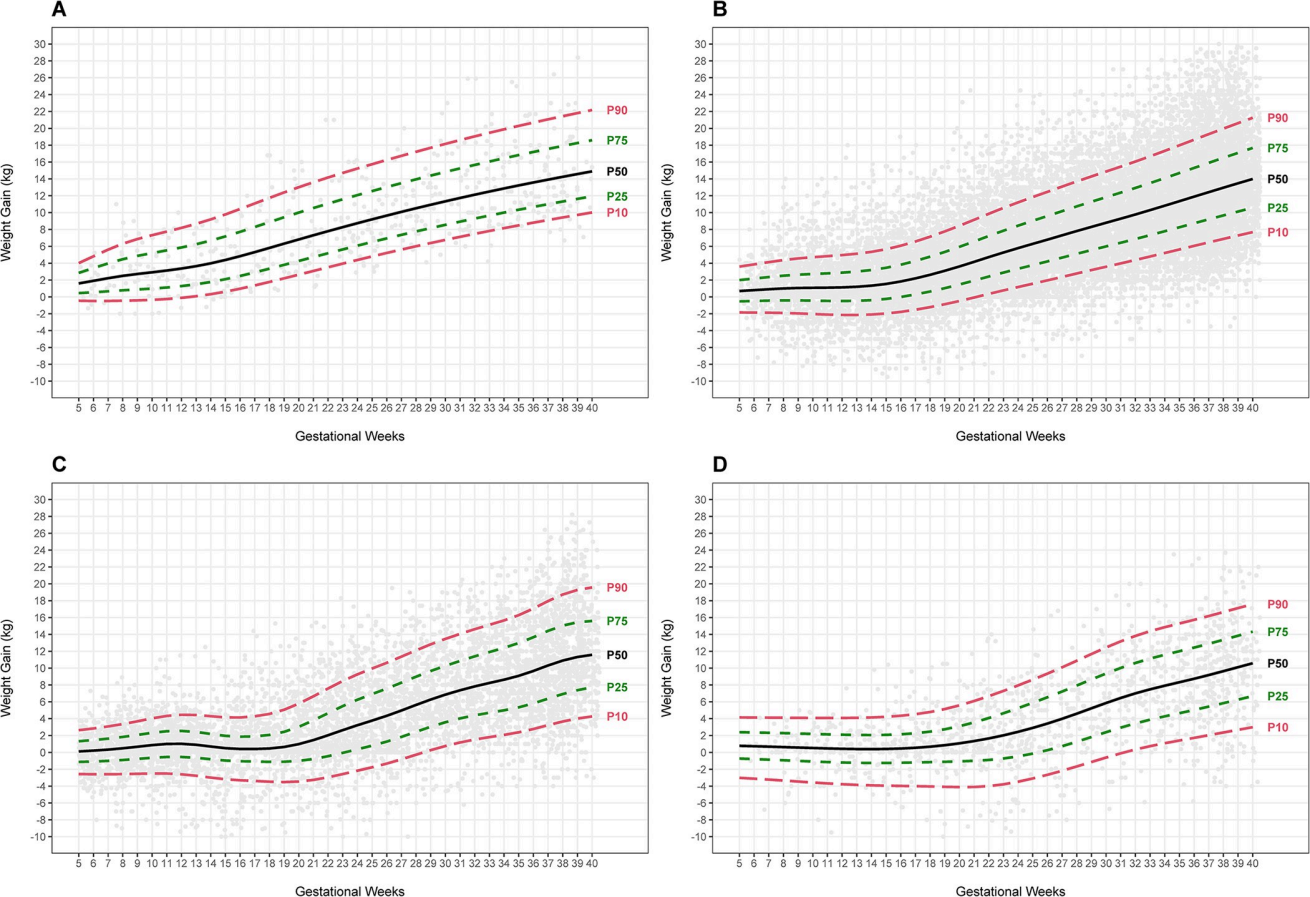
Gestational weight gain curves according to pre-pregnancy BMI for Latin American adolescents. A. underweight (BMI/age <-2 SD; 94 women and 535 records); B. normal-weight (BMI/age ≥ -2 SD and ≤ +1 SD; 4,603 women and 26,273 records); C. overweight (BMI/age > +1 SD and ≤ +2SD; 1,374 women and 6,528 records); D. obesity (BMI/age > +2 SD; 343 women and 1,607 records).

For underweight, positive values for GWG (representing weight gain, not loss) started at the 17^th^ gestational week in the 3^rd^ percentile and sooner (5^th^ week) for the 25^th^, 50^th^, 75^th^, and 97^th^ percentiles. GWG in more advanced weeks presented a gradual and sustained increase (Fig 1A, S2 Table in S1 File). A progressive increase was observed at the 3^rd^ percentile beginning at the 14^th^ gestational week for women with pre-pregnancy normal weight. The 25^th^, 50^th^, 75^th^, and 97^th^ percentiles presented similar behaviors, with small GWG starting at the 5^th^ week and becoming more noticeable from weeks 14–16 (Fig 1B, S3 Table in S1 File).

For overweight, positive values for GWG began at the 19^th^ week for the 3^rd^ percentile. At percentiles 25^th^, 50^th^, and 75^th^, weight losses of a maximum of 1kg were observed between weeks 13 and 19, when GWG was restored. At the 97^th^ percentile, there were always positive values throughout pregnancy (Fig 1C, S4 Table in S1 File). For women with obesity, positive values for GWG began at the 20^th^ gestational week for the 3^rd^ percentile, the 15^th^ week for the 25^th^ and 50^th^ percentiles, and the 16^th^ week for the 75^th^ percentile. In the percentile 97^th^, a progressive gain throughout pregnancy was observed (Fig 1D, S5 Table in S1 File).

The median (P50) GWG differed across the pre-pregnancy BMI categories. The highest values for the 50^th^ percentile in the first, second, and third trimesters were observed for adolescents who started their pregnancy with underweight, followed by normal, overweight, and obesity. The median GWG observed among adolescents with underweight at the end of the first and second trimesters of pregnancy was considerably higher than in adolescents with normal weight. However, at the end of pregnancy, it was similar between both categories (14.9 v. 14.0 kg for underweight and normal weight, respectively) (Table 2).

**Table 2:** Gestational weight gain (kg) at the selected percentiles by trimesters.

	Underweight (< - 2 SD)			Normal weight (≥ -2 SD and ≤ +1 SD)			Overweight (> +1 SD and ≤ +2SD)			Obesity (> + 2 SD)		
Trimester	P25	P50	P75	P25	P50	P75	P25	P50	P75	P25	P50	P75
1^st^ (13 weeks)	1.50	3.64	6.25	-0.47	1.20	3.00	-0.64	0.90	2.42	-1.24	0.40	2.07
2^nd^ (28 weeks)	7.77	10.52	13.95	5.11	7.81	10.74	2.44	5.63	8.98	1.26	4.59	7.93
3^rd^ (40 weeks)	11.93	14.89	18.59	10.57	13.99	17.67	7.74	11.58	15.60	6.68	10.58	14.32

Notes: SD: Standard deviation.

At 40 weeks, it was possible to observe that the median (P50) and IQR (P25 and 75) presented a similar pattern, i.e., the highest values were observed among underweight, followed by normal, overweight, and obesity. The difference between the underweight and obesity categories was 5.2 kg in the 25^th^ percentile (11.9 for underweight v. 6.7 kg for obesity), 4.3 kg in the 50^th^ percentile (14. 9 kg for underweight v. 10.6 for obesity), and 4.3 kg in the 75^th^ percentile (18.6 kg v. 14.3). At 40 weeks, the largest differences between subsequent categories were observed between normal and overweight, for the 25^th^, 50^th^, and 75^th^ percentiles (Table 2).

In the internal validation, the differences between the percentiles estimated in the sample and the theoretical percentiles were close to 1%. For example, for normal weight, we expected 25% of values below the 25^th^ percentile, and the internal validation results were 24.3%. The pre-pregnancy BMI category with the greatest differences between the estimated and expected values was overweight. For the external validation, the differences between observed and expected values were within the 5% accepted difference for normal, overweight, and obesity. For underweight, the differences between observed and expected percentages varied between 0.7% for the 90^th^ percentile and 12.9% for the 50^th^ percentile (Table 3).

**Table 3:** Results of the internal and external validation procedures, data from 6,414 individuals from Latin America.

Pre-pregnancy BMI (SD from WHO charts)	Percentage (%) of observations below/above the selected centiles													
	Internal validation (6,414 individuals and 29,414 records)							External validation (1,684 individuals and 8,879 records)						
	<P3	<P10	<P25	<P50	<P75	<P90	<P97	<P3	<P10	<P25	<P50	<P75	<P90	<P97
Underweight (< - 2 SD)	3.80	9.40	25.00	53.20	75.00	89.60	96.80	6.75	10.97	26.58	62.87	82.70	90.72	94.09
Normal weight (≥ -2 SD and ≤ +1 SD)	2.90	9.73	24.31	50.60	75.30	89.57	96.69	3.34	9.82	23.32	49.83	75.11	89.79	97.03
Overweight (> +1 SD and ≤ +2SD)	3.04	8.94	22.67	50.60	73.15	88.85	96.63	3.78	9.34	23.62	53.36	75.10	90.94	98.17
Obesity (> + 2 SD)	3.74	10.17	22.89	50.24	72.97	90.70	96.74	4.32	9.51	25.94	53.31	72.62	91.64	96.83

Notes: BMI: Body mass index; SD: Standard deviation; WHO: World Health Organization.

## Discussion

This study presents the first GWG charts for Latin American adolescents, according to pre-pregnancy BMI classified using WHO cut-off points for adolescents [13]. The charts should be validated in each country before they are proposed for Health Ministries adoption as monitoring nutritional instruments. The charts were designed using data from apparently healthy women who gave birth to infants at term with adequate weight. The graphs were constructed using GAMLSS, with satisfactory internal and external validation results for almost all pre-pregnancy BMI categories. These charts can potentially help monitor the GWG of pregnant adolescents in routine prenatal care.

There are no international or country-specific guidelines for monitoring weight gain among adolescents. The use of weight gain charts developed for adult women is common worldwide [12]. The most common GWG guideline adopted in several countries, including Latin America, is the 2009 US Institute of Medicine (IOM) guidelines [2]. The IOM suggested classifying the pre-pregnancy BMI of adolescents using the cut-offs established by the WHO for adults. However, this proposal has been questioned because it underestimates the BMI category and recommends greater GWG, which favors postpartum weight retention and the maintenance of excess weight among those women [22,23]. In this sense, classifying the pre-pregnancy BMI of adolescents using the specific WHO charts and cut-offs may be a better approach for any tool for GWG counseling of this group.

Many tools are available for monitoring the GWG of adult individuals in Latin America. One of the first charts used in the Latin American region to evaluate the weight gain of pregnant adults is the one proposed by Mardones and Rosso [24]. This proposal uses cut-off points for BMI before pregnancy that are different from those currently established by the WHO, which does not allow a comparison with the data from our curve.

Another reference used in several Latin American countries is the pregnancy charts proposed by Atalah et al., which establish recommended gain ranges according to the pre-pregnancy BMI category and presents a table and a graph that allows evaluating the behavior of the GWG [25,26]. The authors also designed a theoretical proposal that a pregnant individual with a normal pre-gestational BMI and 1.55 m tall should gain 600 g in the first 10 weeks of pregnancy. Besides, an increase of 20% of the initial weight during pregnancy was recommended since this percentage was associated with lower maternal-fetal morbidity and mortality [26]. For all pre-pregnancy BMI categories, the 25^th^ percentiles of the current charts, for all BMI categories, were close to the lower limit of the weight gain range proposed by Atalah et al. [25,26]. On the other hand, the 75^th^ percentile of our charts corresponded to the Atalah et al. [25,26] upper limit for the underweight category only and was notably higher than the upper limit of those ranges in the other pre-pregnancy BMI categories.

More recently, in 2021, Kac & Carrilho et al. published GWG charts for adult Brazilian women [27]. These charts were developed using data from multiple studies conducted in Brazil, in a similar approach to what we have adopted in this study. When we compare the 25^th^, 50^th^, and 75^th^ percentiles of our charts at 40 weeks with the Brazilian charts, for all pre-pregnancy BMI categories, the GWG values were similar, with the largest difference in the obesity category. At the 50^th^ percentile, underweight adolescents had higher GWG than Brazilian adults in the three trimesters of pregnancy. For the normal-weight category, adults gained more weight than adolescents in the first and second trimesters, and adolescents had greater GWG in the third trimester. Overweight adults had slightly greater gains than adolescents in all trimesters. For obesity, adolescents had lower GWG in the first and higher GWG in the second and third trimesters. These results reinforce the differences between GWG among adults and adolescents and the need for specific tools for the latter.

International GWG standards were developed by the INTERGROWTH-21^st^ study [28]. These standards were created using data from a multicenter study conducted in eight countries, including Brazil as the representant from Latin America. However, they were created only for adult normal-weight women according to first-trimester BMI. When we compare the results of the 25^th^, 50^th^, and 75^th^ percentiles of our charts with the same percentiles of the INTERGROWTH-21^st^ standards for normal-weight individuals at 40 weeks, the values are similar (P25 10.9 kg v. 10.6 kg, P50 13.7 kg v. 14.0 kg, P75 16.9 kg v. 17.7 kg, for INTERGROWTH-21^st^ and our study, respectively). However, the lack of charts for the other BMI categories and the fact that the INTERGROWTH-21^st^ standards do not consider the possibility of GWG in the first trimester of pregnancy makes it challenging to use those standards as a tool for GWG monitoring worldwide, particularly for adolescents.

The values observed in our charts for the 25^th^ and 75^th^ percentiles for individuals with under and normal weight are similar to the GWG ranges recommended in the 2009 IOM guidelines [2]. For underweight, the 25^th^ percentile was 11.9 kg v. 12.5 kg at the IOM lower limit; and the 75^th^ percentile was 18.6 kg v. 18 kg at the IOM upper limit. For normal weight, the 25^th^ percentile was 10.6 kg v. 11.5 kg for the IOM lower limit, and the 75^th^ percentile was 17.7 kg v. 16 kg for the IOM upper limit. For overweight and obesity, the lower limits of the IOM and the 25^th^ percentile of our charts were similar. However, the upper limits of the IOM value are considerably lower than our 75^th^ percentiles. Although the 2009 IOM guidelines are the most established GWG recommendations, they were designed for adult women from the USA. Therefore, they may not apply to contexts where women are shorter or with limited access to health services [2]. Thus, adopting those ranges for monitoring GWG among adolescents living in Latin America may not be appropriate.

It is necessary to highlight that the charts proposed in this study are only a first step towards establishing a comprehensive system to monitor GWG in adolescents. The next phase shall identify optimal GWG cut-offs associated with the lowest maternal and infant adverse outcomes risk. In addition, there is a need to strengthen the information systems of prenatal control programs and the early start of GWG surveillance to contribute to the timely detection and intervention of maternal-perinatal risks among adolescents from Latin American countries.

### Strengths and limitations

The development of GWG monitoring charts for Latin American adolescents is a pioneering initiative consolidating data from nine countries. From this harmonized dataset, a significant number of weight measurements were obtained, with their respective gestational age, which, together with an imputation process in the first trimester, made it possible to build GWG charts according to the four pre-pregnancy BMI categories, starting at the fifth week of gestation. Using WHO charts and cut-offs to classify pre-pregnancy BMI is another important strength. In addition, applying several statistical models with various diagnostic measures and internal and external validations to select the best model is aligned with what is recommended when constructing growth charts [29,30].

As limitations of the study, the insufficient amount of data for underweight adolescents, which resulted in limited satisfactory results in the external validation, needs to be mentioned. In addition, not knowing the origin of pre-pregnancy weight, the lack of a high-quality measurement of gestational age that could be standardized across the datasets, and the lack of information regarding other outcomes, which could have led the charts towards an even more prescriptive approach, are worth mentioning.

On the other hand, the study is influenced by the origin of the data, which impacts the quality and inclusion of records because it relies on secondary data. The data acquired from investigative projects provided complete information that met the study’s criteria. In contrast, data obtained from institutional or national information systems exhibited significant data loss due to missing variables and potential errors in weight records. This introduces a selection bias that could impact the generalizability of the data. Consequently, prior validation within each respective country is imperative before utilizing this data. Besides, the charts apply up to the 40th week of gestation.

## Conclusion

The charts created in this study describe the GWG throughout pregnancy among Latin American adolescents, according to pre-pregnancy BMI, and represent a significant contribution to prenatal control programs. GWG is an indicator that is easy to be understood by women and used by different health professionals. With these new charts, we expect to start changes in the nutritional surveillance of pregnant adolescents, which could positively affect maternal and child health in Latin America during pregnancy and in the medium and long term. The following steps to create final tools to be implemented in prenatal care include defining GWG cut-offs in the graphs based on values associated with lower risks of unfavorable outcomes for the mother-child binomial and disseminating this new tool in the included Latin American countries and others with similar characteristics.

## Supporting information

S1 Fig Flowchart for the constitution of the final dataset. *Notes: Diseases considered: Chronic hypertension or hypertensive disorders during pregnancy, diabetes mellitus or gestational diabetes, tuberculosis, or cardiovascular diseases. Abbreviations: BMI: Body mass index; GA: Gestational age; GWG: Gestational weight gain; HAZ: Height-for-age z score.
(TIF)

S1 File Supplementary tables. (DOCX)
