# Supplementary figures and images for: Gestational weight gain charts for Latin American adolescents
Source: PLoS One. 2023 Nov 1;18(11):e0292070. doi: 10.1371/journal.pone.0292070 (PMC10619863; doi:10.1371/journal.pone.0292070)

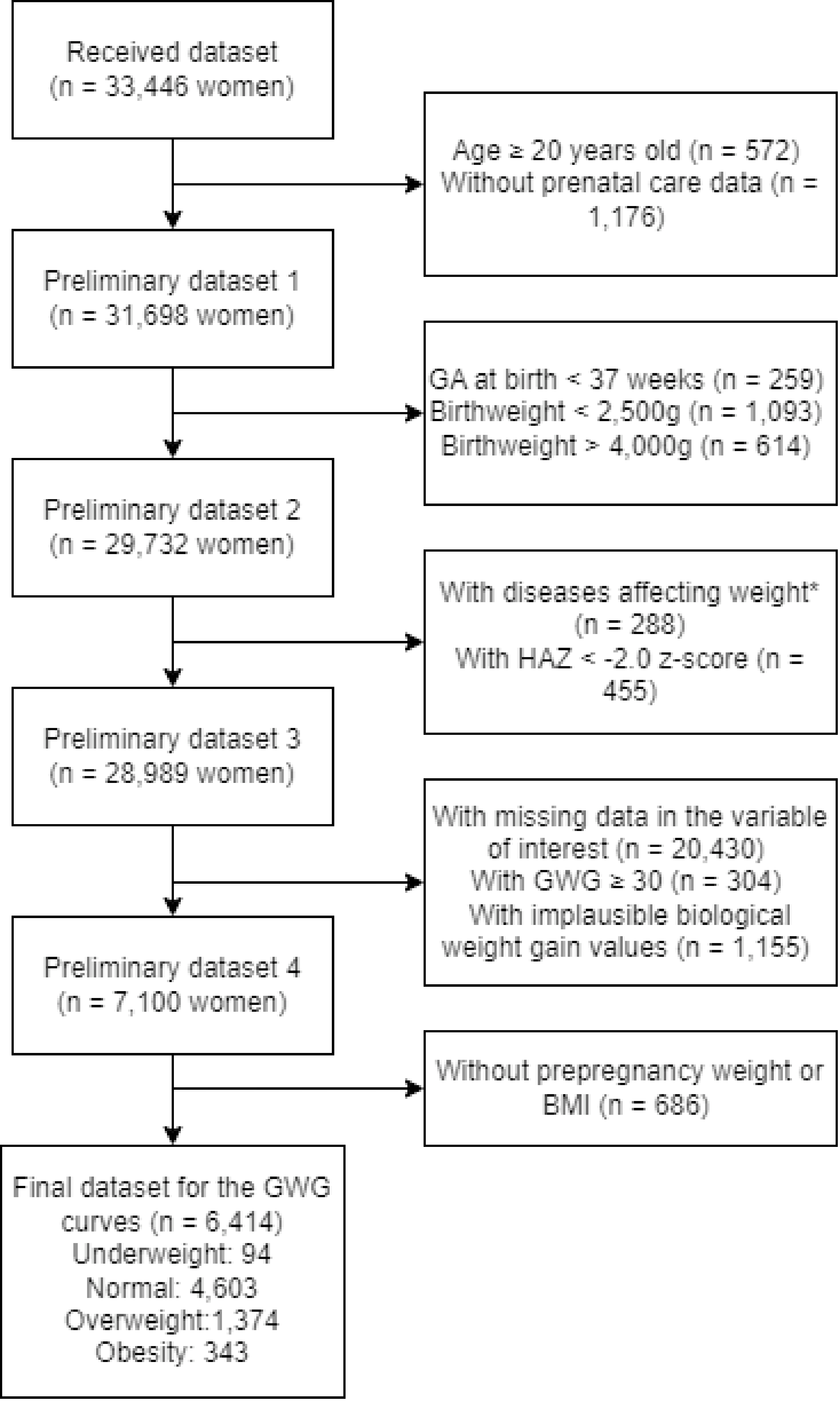

Supplement: S1 Fig — *Notes: Diseases considered: Chronic hypertension or hypertensive disorders during pregnancy, diabetes mellitus or gestational diabetes, tuberculosis, or cardiovascular diseases. Abbreviations: BMI: Body mass index; GA: Gestational age; GWG: Gestational weight gain; HAZ: Height-for-age z score. (TIF) [file pone.0292070.s001.tif]
