# Supplementary material for: Gestational weight gain charts for Latin American adolescents
Source: PLoS One. 2023 Nov 1;18(11):e0292070. doi: 10.1371/journal.pone.0292070 (PMC10619863; doi:10.1371/journal.pone.0292070)
Supplement: S1 File — (DOCX) [file pone.0292070.s002.docx]

**Supplementary tables**

**S1 Table. Number of measures used to construct the tables by pre-pregnancy BMI category** **(including imputed data).**

| Week | Underweight (BMI/age < - 2 SD) | Normal weight (BMI/age ≥ -2 SD and ≤ +1 SD) | Overweight  (BMI/age > +1 SD and ≤ +2SD) | Obesity  (BMI/age (> + 2 SD) | Total |
| --- | --- | --- | --- | --- | --- |
| 5 | 9 | 40 | 124 | 1 | 179 |
| 6 | 12 | 94 | 112 | 6 | 230 |
| 7 | 7 | 138 | 113 | 6 | 271 |
| 8 | 7 | 285 | 197 | 20 | 517 |
| 9 | 13 | 290 | 182 | 18 | 512 |
| 10 | 16 | 369 | 176 | 18 | 589 |
| 11 | 13 | 296 | 179 | 19 | 518 |
| 12 | 11 | 392 | 193 | 17 | 625 |
| 13 | 12 | 341 | 174 | 22 | 562 |
| 14 | 14 | 547 | 127 | 31 | 733 |
| 15 | 14 | 512 | 151 | 31 | 723 |
| 16 | 12 | 561 | 149 | 30 | 768 |
| 17 | 10 | 554 | 143 | 30 | 754 |
| 18 | 13 | 586 | 127 | 32 | 776 |
| 19 | 12 | 573 | 120 | 34 | 758 |
| 20 | 15 | 678 | 206 | 43 | 962 |
| 21 | 13 | 601 | 138 | 38 | 811 |
| 22 | 5 | 705 | 174 | 47 | 953 |
| 23 | 15 | 690 | 177 | 32 | 937 |
| 24 | 18 | 815 | 192 | 58 | 1107 |
| 25 | 23 | 726 | 155 | 53 | 982 |
| 26 | 7 | 600 | 166 | 39 | 838 |
| 27 | 14 | 600 | 152 | 34 | 827 |
| 28 | 16 | 761 | 151 | 31 | 987 |
| 29 | 19 | 567 | 129 | 25 | 769 |
| 30 | 9 | 622 | 155 | 41 | 857 |
| 31 | 15 | 656 | 124 | 29 | 855 |
| 32 | 17 | 789 | 175 | 42 | 1055 |
| 33 | 18 | 719 | 148 | 33 | 951 |
| 34 | 16 | 854 | 208 | 45 | 1157 |
| 35 | 16 | 762 | 181 | 45 | 1039 |
| 36 | 23 | 1048 | 215 | 55 | 1377 |
| 37 | 22 | 1221 | 269 | 74 | 1623 |
| 38 | 25 | 1230 | 301 | 82 | 1676 |
| 39 | 15 | 908 | 251 | 59 | 1272 |
| 40 | 4 | 443 | 149 | 38 | 674 |
| Total | 500 | 21,573 | 6083 | 1258 | 29,414 |

**S2 Table*.* Week-specific model parameters and selected percentiles of gestational weight gain for Latin-American adolescents with pre-pregnancy underweight** (BMI/age < - 2 SD).

| Gestational age (weeks) | Model parameters | | | | Percentiles of gestational weight gain (kg) | | | | | | | | | | |
| --- | --- | --- | --- | --- | --- | --- | --- | --- | --- | --- | --- | --- | --- | --- | --- |
|  | mu | sigma | nu | tau | P2.3 | P3 | P10 | P15.9 | P25 | P50 | P75 | P84.1 | P90 | P97 | P97.7 |
| 5 | 21.60 | 0.08 | -0.54 | 2.25 | -1.40 | -1.26 | -0.46 | -0.05 | 0.45 | 1.60 | 2.85 | 3.46 | 4.01 | 5.19 | 5.42 |
| 6 | 21.90 | 0.09 | -0.61 | 2.35 | -1.54 | -1.38 | -0.49 | -0.03 | 0.55 | 1.90 | 3.41 | 4.16 | 4.81 | 6.24 | 6.52 |
| 7 | 22.22 | 0.10 | -0.67 | 2.44 | -1.61 | -1.44 | -0.48 | 0.03 | 0.67 | 2.22 | 3.98 | 4.84 | 5.61 | 7.27 | 7.60 |
| 8 | 22.50 | 0.11 | -0.73 | 2.53 | -1.64 | -1.46 | -0.45 | 0.09 | 0.78 | 2.50 | 4.47 | 5.45 | 6.31 | 8.18 | 8.55 |
| 9 | 22.72 | 0.12 | -0.77 | 2.61 | -1.63 | -1.45 | -0.41 | 0.16 | 0.88 | 2.72 | 4.87 | 5.93 | 6.86 | 8.90 | 9.30 |
| 10 | 22.92 | 0.12 | -0.81 | 2.69 | -1.58 | -1.40 | -0.35 | 0.23 | 0.98 | 2.92 | 5.20 | 6.33 | 7.32 | 9.48 | 9.91 |
| 11 | 23.12 | 0.13 | -0.85 | 2.77 | -1.50 | -1.32 | -0.25 | 0.34 | 1.11 | 3.12 | 5.52 | 6.70 | 7.75 | 10.01 | 10.45 |
| 12 | 23.36 | 0.13 | -0.88 | 2.83 | -1.37 | -1.19 | -0.11 | 0.49 | 1.28 | 3.36 | 5.86 | 7.10 | 8.19 | 10.54 | 11.01 |
| 13 | 23.64 | 0.13 | -0.92 | 2.89 | -1.19 | -1.01 | 0.08 | 0.69 | 1.50 | 3.64 | 6.25 | 7.53 | 8.66 | 11.11 | 11.59 |
| 14 | 23.98 | 0.14 | -0.95 | 2.93 | -0.96 | -0.78 | 0.32 | 0.94 | 1.77 | 3.98 | 6.68 | 8.02 | 9.20 | 11.74 | 12.24 |
| 15 | 24.38 | 0.14 | -0.97 | 2.97 | -0.68 | -0.50 | 0.62 | 1.25 | 2.10 | 4.38 | 7.18 | 8.56 | 9.79 | 12.43 | 12.95 |
| 16 | 24.83 | 0.14 | -1.00 | 2.99 | -0.35 | -0.16 | 0.97 | 1.62 | 2.48 | 4.83 | 7.72 | 9.16 | 10.42 | 13.17 | 13.71 |
| 17 | 25.31 | 0.14 | -1.02 | 3.01 | 0.02 | 0.21 | 1.36 | 2.02 | 2.90 | 5.31 | 8.29 | 9.78 | 11.09 | 13.93 | 14.49 |
| 18 | 25.81 | 0.14 | -1.04 | 3.02 | 0.41 | 0.60 | 1.77 | 2.45 | 3.35 | 5.81 | 8.87 | 10.40 | 11.75 | 14.68 | 15.26 |
| 19 | 26.32 | 0.14 | -1.07 | 3.03 | 0.81 | 1.01 | 2.20 | 2.89 | 3.80 | 6.32 | 9.44 | 11.01 | 12.39 | 15.41 | 16.00 |
| 20 | 26.82 | 0.14 | -1.09 | 3.03 | 1.23 | 1.43 | 2.  64 | 3.33 | 4.26 | 6.82 | 10.00 | 11.60 | 13.01 | 16.09 | 16.70 |
| 21 | 27.31 | 0.14 | -1.10 | 3.03 | 1.65 | 1.85 | 3.08 | 3.78 | 4.72 | 7.31 | 10.55 | 12.17 | 13.60 | 16.74 | 17.36 |
| 22 | 27.80 | 0.14 | -1.12 | 3.03 | 2.07 | 2.28 | 3.51 | 4.23 | 5.18 | 7.80 | 11.07 | 12.71 | 14.17 | 17.35 | 17.98 |
| 23 | 28.27 | 0.14 | -1.14 | 3.02 | 2.49 | 2.70 | 3.95 | 4.67 | 5.63 | 8.27 | 11.58 | 13.24 | 14.71 | 17.93 | 18.57 |
| 24 | 28.74 | 0.14 | -1.16 | 3.02 | 2.91 | 3.12 | 4.38 | 5.10 | 6.08 | 8.74 | 12.07 | 13.75 | 15.23 | 18.49 | 19.13 |
| 25 | 29.20 | 0.14 | -1.17 | 3.01 | 3.31 | 3.53 | 4.80 | 5.53 | 6.51 | 9.20 | 12.56 | 14.24 | 15.74 | 19.03 | 19.68 |
| 26 | 29.65 | 0.14 | -1.19 | 3.00 | 3.71 | 3.93 | 5.21 | 5.95 | 6.94 | 9.65 | 13.03 | 14.73 | 16.24 | 19.56 | 20.22 |
| 27 | 30.09 | 0.13 | -1.20 | 2.99 | 4.10 | 4.32 | 5.62 | 6.36 | 7.36 | 10.09 | 13.49 | 15.21 | 16.74 | 20.09 | 20.76 |
| 28 | 30.52 | 0.13 | -1.22 | 2.98 | 4.48 | 4.70 | 6.01 | 6.76 | 7.77 | 10.52 | 13.95 | 15.68 | 17.22 | 20.61 | 21.28 |
| 29 | 30.93 | 0.13 | -1.23 | 2.98 | 4.85 | 5.07 | 6.39 | 7.15 | 8.16 | 10.93 | 14.39 | 16.14 | 17.69 | 21.12 | 21.80 |
| 30 | 31.34 | 0.13 | -1.24 | 2.97 | 5.20 | 5.42 | 6.76 | 7.53 | 8.55 | 11.34 | 14.83 | 16.59 | 18.15 | 21.62 | 22.31 |
| 31 | 31.74 | 0.13 | -1.26 | 2.96 | 5.55 | 5.78 | 7.12 | 7.90 | 8.93 | 11.74 | 15.25 | 17.02 | 18.60 | 22.10 | 22.80 |
| 32 | 32.12 | 0.13 | -1.27 | 2.95 | 5.89 | 6.12 | 7.48 | 8.26 | 9.30 | 12.12 | 15.66 | 17.45 | 19.04 | 22.58 | 23.28 |
| 33 | 32.50 | 0.13 | -1.28 | 2.94 | 6.22 | 6.45 | 7.82 | 8.61 | 9.65 | 12.50 | 16.06 | 17.86 | 19.47 | 23.04 | 23.75 |
| 34 | 32.87 | 0.13 | -1.29 | 2.93 | 6.54 | 6.77 | 8.16 | 8.95 | 10.00 | 12.87 | 16.45 | 18.26 | 19.89 | 23.49 | 24.21 |
| 35 | 33.23 | 0.13 | -1.31 | 2.93 | 6.86 | 7.09 | 8.49 | 9.28 | 10.34 | 13.23 | 16.83 | 18.65 | 20.29 | 23.92 | 24.65 |
| 36 | 33.57 | 0.13 | -1.32 | 2.92 | 7.17 | 7.40 | 8.81 | 9.61 | 10.68 | 13.57 | 17.20 | 19.04 | 20.68 | 24.34 | 25.08 |
| 37 | 33.91 | 0.13 | -1.33 | 2.91 | 7.47 | 7.70 | 9.12 | 9.93 | 11.00 | 13.91 | 17.56 | 19.41 | 21.07 | 24.76 | 25.50 |
| 38 | 34.25 | 0.13 | -1.34 | 2.90 | 7.76 | 8.00 | 9.42 | 10.24 | 11.32 | 14.25 | 17.91 | 19.77 | 21.44 | 25.16 | 25.91 |
| 39 | 34.57 | 0.13 | -1.35 | 2.90 | 8.05 | 8.29 | 9.72 | 10.54 | 11.63 | 14.57 | 18.25 | 20.13 | 21.81 | 25.56 | 26.31 |
| 40 | 34.89 | 0.13 | -1.36 | 2.89 | 8.33 | 8.57 | 10.02 | 10.84 | 11.93 | 14.89 | 18.59 | 20.47 | 22.17 | 25.94 | 26.71 |

**S3 Table*.* Week-specific model parameters and selected percentiles of gestational weight gain for Latin-American adolescents with pre-pregnancy normal weight** (BMI/age ≥ -2 SD and ≤ +1 SD).

| Gestational age (weeks) | Model parameters | | | | Percentiles of gestational weight gain (kg) | | | | | | | | | | |
| --- | --- | --- | --- | --- | --- | --- | --- | --- | --- | --- | --- | --- | --- | --- | --- |
|  | mu | sigma | nu | tau | P2.3 | P3 | P10 | P15.9 | P25 | P50 | P75 | P84.1 | P90 | P97 | P97.7 |
| 5 | 20.69 | 0.08 | -0.02 | 2.94 | -4.11 | -3.66 | -1.84 | -1.19 | -0.53 | 0.69 | 2.00 | 2.77 | 3.59 | 6.25 | 6.99 |
| 6 | 20.80 | 0.08 | -0.01 | 2.98 | -4.21 | -3.75 | -1.86 | -1.17 | -0.48 | 0.80 | 2.18 | 2.99 | 3.86 | 6.65 | 7.43 |
| 7 | 20.91 | 0.09 | 0.00 | 3.02 | -4.31 | -3.83 | -1.88 | -1.16 | -0.44 | 0.91 | 2.36 | 3.21 | 4.12 | 7.04 | 7.86 |
| 8 | 21.00 | 0.09 | 0.01 | 3.06 | -4.41 | -3.92 | -1.90 | -1.16 | -0.41 | 1.00 | 2.52 | 3.41 | 4.37 | 7.41 | 8.26 |
| 9 | 21.06 | 0.10 | 0.02 | 3.12 | -4.52 | -4.02 | -1.96 | -1.19 | -0.41 | 1.06 | 2.64 | 3.57 | 4.56 | 7.71 | 8.58 |
| 10 | 21.08 | 0.10 | 0.03 | 3.20 | -4.63 | -4.13 | -2.03 | -1.25 | -0.45 | 1.08 | 2.72 | 3.69 | 4.71 | 7.92 | 8.81 |
| 11 | 21.10 | 0.10 | 0.04 | 3.32 | -4.70 | -4.21 | -2.10 | -1.30 | -0.48 | 1.10 | 2.79 | 3.78 | 4.83 | 8.08 | 8.96 |
| 12 | 21.13 | 0.11 | 0.05 | 3.48 | -4.74 | -4.25 | -2.14 | -1.33 | -0.49 | 1.13 | 2.88 | 3.90 | 4.96 | 8.22 | 9.09 |
| 13 | 21.20 | 0.11 | 0.07 | 3.68 | -4.72 | -4.24 | -2.14 | -1.32 | -0.47 | 1.20 | 3.00 | 4.04 | 5.12 | 8.37 | 9.22 |
| 14 | 21.33 | 0.11 | 0.07 | 3.93 | -4.65 | -4.18 | -2.09 | -1.26 | -0.39 | 1.33 | 3.19 | 4.25 | 5.35 | 8.57 | 9.40 |
| 15 | 21.54 | 0.12 | 0.08 | 4.24 | -4.51 | -4.05 | -1.97 | -1.13 | -0.24 | 1.54 | 3.45 | 4.54 | 5.66 | 8.86 | 9.67 |
| 16 | 21.83 | 0.12 | 0.09 | 4.60 | -4.30 | -3.85 | -1.78 | -0.93 | -0.01 | 1.83 | 3.82 | 4.94 | 6.07 | 9.26 | 10.06 |
| 17 | 22.20 | 0.12 | 0.10 | 5.04 | -4.04 | -3.60 | -1.52 | -0.65 | 0.29 | 2.20 | 4.26 | 5.42 | 6.57 | 9.76 | 10.54 |
| 18 | 22.62 | 0.13 | 0.11 | 5.55 | -3.74 | -3.30 | -1.22 | -0.33 | 0.64 | 2.62 | 4.77 | 5.96 | 7.14 | 10.33 | 11.10 |
| 19 | 23.10 | 0.13 | 0.12 | 6.15 | -3.40 | -2.96 | -0.87 | 0.03 | 1.03 | 3.10 | 5.33 | 6.56 | 7.77 | 10.97 | 11.72 |
| 20 | 23.61 | 0.13 | 0.13 | 6.85 | -3.03 | -2.59 | -0.49 | 0.44 | 1.47 | 3.61 | 5.94 | 7.21 | 8.44 | 11.65 | 12.40 |
| 21 | 24.16 | 0.14 | 0.14 | 7.64 | -2.63 | -2.20 | -0.07 | 0.87 | 1.94 | 4.16 | 6.58 | 7.88 | 9.15 | 12.38 | 13.12 |
| 22 | 24.72 | 0.14 | 0.15 | 8.52 | -2.23 | -1.79 | 0.35 | 1.32 | 2.42 | 4.72 | 7.22 | 8.57 | 9.86 | 13.12 | 13.85 |
| 23 | 25.27 | 0.14 | 0.16 | 9.50 | -1.83 | -1.40 | 0.77 | 1.76 | 2.88 | 5.27 | 7.85 | 9.24 | 10.55 | 13.84 | 14.57 |
| 24 | 25.79 | 0.14 | 0.17 | 10.58 | -1.45 | -1.02 | 1.17 | 2.18 | 3.33 | 5.79 | 8.45 | 9.87 | 11.21 | 14.52 | 15.25 |
| 25 | 26.29 | 0.14 | 0.17 | 11.76 | -1.09 | -0.65 | 1.56 | 2.59 | 3.77 | 6.29 | 9.03 | 10.48 | 11.84 | 15.17 | 15.90 |
| 26 | 26.79 | 0.14 | 0.18 | 13.05 | -0.72 | -0.28 | 1.96 | 3.01 | 4.21 | 6.79 | 9.60 | 11.08 | 12.46 | 15.82 | 16.54 |
| 27 | 27.30 | 0.15 | 0.19 | 14.48 | -0.33 | 0.10 | 2.37 | 3.43 | 4.66 | 7.30 | 10.17 | 11.68 | 13.09 | 16.46 | 17.18 |
| 28 | 27.81 | 0.15 | 0.20 | 16.06 | 0.05 | 0.49 | 2.78 | 3.86 | 5.11 | 7.81 | 10.74 | 12.27 | 13.70 | 17.09 | 17.81 |
| 29 | 28.31 | 0.15 | 0.21 | 17.82 | 0.43 | 0.87 | 3.18 | 4.28 | 5.55 | 8.31 | 11.29 | 12.85 | 14.29 | 17.70 | 18.42 |
| 30 | 28.79 | 0.15 | 0.22 | 19.79 | 0.80 | 1.24 | 3.58 | 4.69 | 5.99 | 8.79 | 11.83 | 13.41 | 14.87 | 18.30 | 19.02 |
| 31 | 29.28 | 0.15 | 0.22 | 21.98 | 1.17 | 1.62 | 3.98 | 5.11 | 6.42 | 9.28 | 12.37 | 13.98 | 15.46 | 18.91 | 19.62 |
| 32 | 29.78 | 0.15 | 0.23 | 24.42 | 1.54 | 1.99 | 4.38 | 5.53 | 6.87 | 9.78 | 12.93 | 14.56 | 16.06 | 19.53 | 20.25 |
| 33 | 30.29 | 0.15 | 0.24 | 27.15 | 1.91 | 2.37 | 4.79 | 5.96 | 7.32 | 10.29 | 13.50 | 15.16 | 16.68 | 20.19 | 20.91 |
| 34 | 30.82 | 0.15 | 0.25 | 30.20 | 2.29 | 2.75 | 5.21 | 6.40 | 7.79 | 10.82 | 14.09 | 15.78 | 17.33 | 20.88 | 21.60 |
| 35 | 31.36 | 0.15 | 0.26 | 33.61 | 2.66 | 3.13 | 5.63 | 6.84 | 8.26 | 11.36 | 14.69 | 16.41 | 17.98 | 21.58 | 22.31 |
| 36 | 31.89 | 0.15 | 0.26 | 37.40 | 3.04 | 3.51 | 6.05 | 7.28 | 8.73 | 11.89 | 15.30 | 17.05 | 18.65 | 22.29 | 23.03 |
| 37 | 32.43 | 0.15 | 0.27 | 41.62 | 3.40 | 3.89 | 6.47 | 7.73 | 9.20 | 12.43 | 15.90 | 17.68 | 19.31 | 23.00 | 23.75 |
| 38 | 32.96 | 0.15 | 0.28 | 46.30 | 3.77 | 4.26 | 6.88 | 8.16 | 9.67 | 12.96 | 16.50 | 18.31 | 19.97 | 23.71 | 24.47 |
| 39 | 33.48 | 0.15 | 0.29 | 51.48 | 4.12 | 4.62 | 7.28 | 8.59 | 10.12 | 13.48 | 17.09 | 18.93 | 20.61 | 24.41 | 25.18 |
| 40 | 33.99 | 0.15 | 0.29 | 57.21 | 4.47 | 4.97 | 7.68 | 9.01 | 10.57 | 13.99 | 17.67 | 19.55 | 21.25 | 25.10 | 25.88 |

**S4 Table*.* Week-specific model parameters and selected percentiles of gestational weight gain for Latin-American adolescents with pre-pregnancy overweight** (BMI/age ≥ +1 SD and ≤ +2SD).

| Gestational age (weeks) | Model parameters | | | | Percentiles of gestational weight gain (kg) | | | | | | | | | | |
| --- | --- | --- | --- | --- | --- | --- | --- | --- | --- | --- | --- | --- | --- | --- | --- |
|  | mu | sigma | nu | tau | P2.3 | P3 | P10 | P15.9 | P25 | P50 | P75 | P84.1 | P90 | P97 | P97.7 |
| 5 | 20.12 | 0.11 | 1.49 | 1.31 | -4.71 | -4.33 | -2.57 | -1.85 | -1.13 | 0.12 | 1.32 | 2.00 | 2.63 | 4.12 | 4.42 |
| 6 | 20.21 | 0.11 | 1.47 | 1.29 | -4.85 | -4.45 | -2.59 | -1.84 | -1.08 | 0.21 | 1.46 | 2.17 | 2.84 | 4.41 | 4.73 |
| 7 | 20.33 | 0.12 | 1.45 | 1.26 | -5.00 | -4.57 | -2.59 | -1.80 | -1.01 | 0.33 | 1.63 | 2.37 | 3.08 | 4.75 | 5.09 |
| 8 | 20.49 | 0.12 | 1.43 | 1.23 | -5.13 | -4.68 | -2.57 | -1.74 | -0.90 | 0.49 | 1.84 | 2.62 | 3.37 | 5.14 | 5.51 |
| 9 | 20.67 | 0.13 | 1.40 | 1.20 | -5.27 | -4.78 | -2.54 | -1.66 | -0.77 | 0.67 | 2.08 | 2.90 | 3.69 | 5.59 | 5.99 |
| 10 | 20.86 | 0.13 | 1.37 | 1.16 | -5.43 | -4.90 | -2.50 | -1.57 | -0.64 | 0.86 | 2.33 | 3.19 | 4.04 | 6.08 | 6.51 |
| 11 | 21.00 | 0.14 | 1.34 | 1.12 | -5.64 | -5.07 | -2.51 | -1.52 | -0.54 | 1.00 | 2.51 | 3.43 | 4.33 | 6.54 | 7.00 |
| 12 | 21.02 | 0.15 | 1.29 | 1.05 | -5.97 | -5.35 | -2.60 | -1.56 | -0.54 | 1.02 | 2.55 | 3.51 | 4.46 | 6.87 | 7.39 |
| 13 | 20.90 | 0.15 | 1.23 | 0.97 | -6.37 | -5.70 | -2.77 | -1.68 | -0.64 | 0.90 | 2.42 | 3.42 | 4.44 | 7.07 | 7.65 |
| 14 | 20.70 | 0.16 | 1.16 | 0.89 | -6.79 | -6.08 | -2.99 | -1.86 | -0.81 | 0.70 | 2.19 | 3.22 | 4.29 | 7.16 | 7.81 |
| 15 | 20.50 | 0.17 | 1.08 | 0.82 | -7.15 | -6.41 | -3.19 | -2.03 | -0.97 | 0.50 | 1.96 | 3.02 | 4.15 | 7.27 | 7.98 |
| 16 | 20.41 | 0.17 | 0.99 | 0.78 | -7.38 | -6.62 | -3.31 | -2.13 | -1.05 | 0.41 | 1.87 | 2.95 | 4.14 | 7.50 | 8.29 |
| 17 | 20.40 | 0.18 | 0.90 | 0.77 | -7.50 | -6.73 | -3.39 | -2.19 | -1.08 | 0.40 | 1.90 | 3.03 | 4.28 | 7.85 | 8.69 |
| 18 | 20.46 | 0.19 | 0.80 | 0.81 | -7.52 | -6.77 | -3.48 | -2.26 | -1.12 | 0.46 | 2.07 | 3.26 | 4.56 | 8.27 | 9.15 |
| 19 | 20.64 | 0.19 | 0.72 | 0.89 | -7.39 | -6.69 | -3.52 | -2.29 | -1.11 | 0.64 | 2.44 | 3.70 | 5.06 | 8.82 | 9.69 |
| 20 | 20.98 | 0.19 | 0.65 | 1.04 | -7.08 | -6.45 | -3.46 | -2.24 | -1.00 | 0.98 | 3.03 | 4.38 | 5.78 | 9.50 | 10.33 |
| 21 | 21.46 | 0.19 | 0.59 | 1.23 | -6.63 | -6.06 | -3.27 | -2.06 | -0.78 | 1.46 | 3.80 | 5.23 | 6.65 | 10.25 | 11.04 |
| 22 | 22.04 | 0.19 | 0.56 | 1.44 | -6.09 | -5.58 | -2.95 | -1.76 | -0.45 | 2.04 | 4.65 | 6.15 | 7.58 | 11.04 | 11.77 |
| 23 | 22.64 | 0.19 | 0.54 | 1.64 | -5.56 | -5.09 | -2.57 | -1.39 | -0.05 | 2.64 | 5.49 | 7.03 | 8.47 | 11.81 | 12.49 |
| 24 | 23.22 | 0.19 | 0.54 | 1.81 | -5.08 | -4.62 | -2.17 | -0.99 | 0.37 | 3.22 | 6.25 | 7.82 | 9.26 | 12.51 | 13.16 |
| 25 | 23.77 | 0.19 | 0.56 | 1.92 | -4.64 | -4.19 | -1.77 | -0.58 | 0.80 | 3.77 | 6.92 | 8.51 | 9.95 | 13.14 | 13.77 |
| 26 | 24.34 | 0.19 | 0.58 | 1.99 | -4.22 | -3.77 | -1.33 | -0.13 | 1.28 | 4.34 | 7.57 | 9.19 | 10.63 | 13.79 | 14.40 |
| 27 | 24.97 | 0.19 | 0.60 | 2.03 | -3.76 | -3.30 | -0.82 | 0.40 | 1.84 | 4.97 | 8.27 | 9.91 | 11.36 | 14.52 | 15.14 |
| 28 | 25.63 | 0.19 | 0.62 | 2.02 | -3.28 | -2.81 | -0.28 | 0.97 | 2.44 | 5.63 | 8.98 | 10.64 | 12.11 | 15.30 | 15.92 |
| 29 | 26.27 | 0.19 | 0.64 | 2.00 | -2.83 | -2.34 | 0.27 | 1.54 | 3.04 | 6.27 | 9.66 | 11.33 | 12.82 | 16.06 | 16.69 |
| 30 | 26.85 | 0.18 | 0.66 | 2.00 | -2.40 | -1.91 | 0.76 | 2.06 | 3.58 | 6.85 | 10.27 | 11.97 | 13.47 | 16.73 | 17.37 |
| 31 | 27.37 | 0.18 | 0.67 | 2.04 | -2.00 | -1.50 | 1.18 | 2.49 | 4.03 | 7.37 | 10.85 | 12.55 | 14.06 | 17.31 | 17.94 |
| 32 | 27.83 | 0.18 | 0.69 | 2.14 | -1.62 | -1.13 | 1.53 | 2.84 | 4.40 | 7.83 | 11.40 | 13.12 | 14.62 | 17.81 | 18.43 |
| 33 | 28.24 | 0.18 | 0.70 | 2.26 | -1.29 | -0.81 | 1.81 | 3.13 | 4.71 | 8.24 | 11.91 | 13.64 | 15.14 | 18.27 | 18.86 |
| 34 | 28.63 | 0.18 | 0.70 | 2.38 | -0.99 | -0.52 | 2.08 | 3.40 | 4.99 | 8.63 | 12.41 | 14.16 | 15.66 | 18.75 | 19.33 |
| 35 | 29.09 | 0.18 | 0.70 | 2.45 | -0.70 | -0.23 | 2.38 | 3.72 | 5.34 | 9.09 | 12.98 | 14.76 | 16.28 | 19.39 | 19.96 |
| 36 | 29.66 | 0.19 | 0.70 | 2.46 | -0.38 | 0.10 | 2.78 | 4.14 | 5.81 | 9.66 | 13.67 | 15.51 | 17.07 | 20.26 | 20.86 |
| 37 | 30.32 | 0.19 | 0.69 | 2.39 | -0.06 | 0.45 | 3.23 | 4.66 | 6.38 | 10.32 | 14.42 | 16.33 | 17.96 | 21.32 | 21.95 |
| 38 | 30.90 | 0.19 | 0.67 | 2.23 | 0.17 | 0.71 | 3.67 | 5.15 | 6.92 | 10.90 | 15.04 | 17.02 | 18.73 | 22.33 | 23.02 |
| 39 | 31.32 | 0.19 | 0.65 | 2.02 | 0.27 | 0.86 | 4.01 | 5.56 | 7.38 | 11.32 | 15.44 | 17.47 | 19.27 | 23.18 | 23.94 |
| 40 | 31.58 | 0.19 | 0.63 | 1.79 | 0.24 | 0.88 | 4.27 | 5.89 | 7.74 | 11.58 | 15.60 | 17.68 | 19.57 | 23.83 | 24.68 |

**S5 Table*.* Week-specific model parameters and selected percentiles of gestational weight gain for Latin-American adolescents with pre-pregnancy obesity** (BMI/age > + 2SD).

| Gestational age (weeks) | Model parameters | | | | Percentiles of gestational weight gain (kg) | | | | | | | | | | |
| --- | --- | --- | --- | --- | --- | --- | --- | --- | --- | --- | --- | --- | --- | --- | --- |
|  | mu | sigma | nu | tau | P2.3 | P3 | P10 | P15.9 | P25 | P50 | P75 | P84.1 | P90 | P97 | P97.7 |
| 5 | 20.45 | 0.22 | 4.85 | 0.86 | -6.87 | -6.20 | -3.02 | -1.83 | -0.73 | 0.78 | 2.40 | 3.31 | 4.15 | 5.99 | 6.34 |
| 6 | 20.42 | 0.22 | 4.70 | 0.86 | -7.03 | -6.35 | -3.12 | -1.91 | -0.79 | 0.73 | 2.36 | 3.29 | 4.13 | 6.01 | 6.38 |
| 7 | 20.39 | 0.22 | 4.53 | 0.86 | -7.21 | -6.51 | -3.23 | -1.99 | -0.86 | 0.68 | 2.32 | 3.26 | 4.12 | 6.04 | 6.42 |
| 8 | 20.35 | 0.21 | 4.36 | 0.86 | -7.40 | -6.69 | -3.34 | -2.08 | -0.93 | 0.63 | 2.28 | 3.23 | 4.11 | 6.08 | 6.46 |
| 9 | 20.32 | 0.21 | 4.17 | 0.86 | -7.60 | -6.88 | -3.46 | -2.18 | -1.01 | 0.58 | 2.24 | 3.20 | 4.10 | 6.12 | 6.52 |
| 10 | 20.29 | 0.21 | 3.98 | 0.86 | -7.81 | -7.07 | -3.57 | -2.27 | -1.08 | 0.52 | 2.19 | 3.17 | 4.09 | 6.17 | 6.59 |
| 11 | 20.26 | 0.21 | 3.77 | 0.85 | -8.01 | -7.25 | -3.68 | -2.35 | -1.15 | 0.47 | 2.14 | 3.14 | 4.08 | 6.24 | 6.67 |
| 12 | 20.24 | 0.21 | 3.56 | 0.84 | -8.21 | -7.43 | -3.77 | -2.42 | -1.20 | 0.43 | 2.10 | 3.11 | 4.08 | 6.33 | 6.78 |
| 13 | 20.24 | 0.21 | 3.34 | 0.83 | -8.41 | -7.60 | -3.85 | -2.48 | -1.24 | 0.40 | 2.07 | 3.10 | 4.10 | 6.45 | 6.93 |
| 14 | 20.26 | 0.21 | 3.12 | 0.82 | -8.59 | -7.76 | -3.91 | -2.51 | -1.25 | 0.39 | 2.06 | 3.11 | 4.14 | 6.60 | 7.10 |
| 15 | 20.30 | 0.21 | 2.89 | 0.81 | -8.76 | -7.90 | -3.95 | -2.53 | -1.25 | 0.41 | 2.08 | 3.16 | 4.22 | 6.79 | 7.32 |
| 16 | 20.37 | 0.21 | 2.67 | 0.81 | -8.92 | -8.03 | -3.98 | -2.53 | -1.23 | 0.46 | 2.15 | 3.25 | 4.35 | 7.03 | 7.59 |
| 17 | 20.47 | 0.21 | 2.46 | 0.83 | -9.07 | -8.16 | -4.01 | -2.52 | -1.20 | 0.55 | 2.27 | 3.40 | 4.53 | 7.33 | 7.91 |
| 18 | 20.62 | 0.21 | 2.25 | 0.86 | -9.22 | -8.28 | -4.04 | -2.53 | -1.16 | 0.68 | 2.46 | 3.63 | 4.79 | 7.67 | 8.28 |
| 19 | 20.80 | 0.21 | 2.05 | 0.91 | -9.33 | -8.38 | -4.08 | -2.53 | -1.11 | 0.85 | 2.74 | 3.94 | 5.14 | 8.08 | 8.70 |
| 20 | 21.03 | 0.21 | 1.86 | 1.00 | -9.36 | -8.41 | -4.12 | -2.54 | -1.07 | 1.07 | 3.11 | 4.36 | 5.58 | 8.54 | 9.16 |
| 21 | 21.32 | 0.20 | 1.69 | 1.12 | -9.22 | -8.30 | -4.10 | -2.52 | -1.00 | 1.34 | 3.56 | 4.85 | 6.10 | 9.04 | 9.65 |
| 22 | 21.64 | 0.20 | 1.53 | 1.28 | -8.85 | -7.98 | -4.00 | -2.45 | -0.89 | 1.66 | 4.07 | 5.41 | 6.67 | 9.57 | 10.16 |
| 23 | 22.02 | 0.20 | 1.40 | 1.46 | -8.25 | -7.46 | -3.79 | -2.28 | -0.72 | 2.02 | 4.64 | 6.03 | 7.29 | 10.14 | 10.70 |
| 24 | 22.44 | 0.20 | 1.28 | 1.65 | -7.54 | -6.84 | -3.48 | -2.03 | -0.47 | 2.44 | 5.25 | 6.67 | 7.95 | 10.74 | 11.28 |
| 25 | 22.90 | 0.20 | 1.18 | 1.82 | -6.84 | -6.21 | -3.09 | -1.69 | -0.14 | 2.91 | 5.88 | 7.34 | 8.63 | 11.40 | 11.93 |
| 26 | 23.42 | 0.20 | 1.10 | 1.95 | -6.19 | -5.62 | -2.66 | -1.29 | 0.26 | 3.42 | 6.54 | 8.04 | 9.35 | 12.11 | 12.64 |
| 27 | 23.99 | 0.20 | 1.04 | 2.04 | -5.60 | -5.05 | -2.18 | -0.82 | 0.73 | 3.99 | 7.22 | 8.76 | 10.09 | 12.89 | 13.43 |
| 28 | 24.59 | 0.20 | 0.99 | 2.09 | -5.05 | -4.51 | -1.67 | -0.31 | 1.26 | 4.59 | 7.93 | 9.51 | 10.87 | 13.73 | 14.27 |
| 29 | 25.23 | 0.20 | 0.96 | 2.12 | -4.52 | -3.98 | -1.14 | 0.23 | 1.83 | 5.23 | 8.65 | 10.27 | 11.67 | 14.60 | 15.15 |
| 30 | 25.86 | 0.20 | 0.93 | 2.12 | -4.02 | -3.48 | -0.61 | 0.78 | 2.39 | 5.86 | 9.36 | 11.02 | 12.45 | 15.45 | 16.02 |
| 31 | 26.46 | 0.20 | 0.91 | 2.11 | -3.57 | -3.03 | -0.11 | 1.30 | 2.94 | 6.46 | 10.02 | 11.71 | 13.18 | 16.25 | 16.84 |
| 32 | 27.00 | 0.19 | 0.91 | 2.10 | -3.18 | -2.62 | 0.34 | 1.77 | 3.44 | 7.00 | 10.61 | 12.33 | 13.82 | 16.96 | 17.56 |
| 33 | 27.48 | 0.19 | 0.91 | 2.08 | -2.84 | -2.28 | 0.74 | 2.19 | 3.88 | 7.48 | 11.13 | 12.87 | 14.38 | 17.56 | 18.17 |
| 34 | 27.91 | 0.19 | 0.93 | 2.07 | -2.57 | -1.99 | 1.09 | 2.56 | 4.28 | 7.91 | 11.58 | 13.33 | 14.86 | 18.07 | 18.68 |
| 35 | 28.32 | 0.19 | 0.97 | 2.05 | -2.34 | -1.75 | 1.41 | 2.91 | 4.65 | 8.32 | 12.01 | 13.77 | 15.29 | 18.52 | 19.13 |
| 36 | 28.74 | 0.19 | 1.01 | 2.03 | -2.15 | -1.53 | 1.72 | 3.26 | 5.03 | 8.74 | 12.43 | 14.19 | 15.73 | 18.95 | 19.57 |
| 37 | 29.18 | 0.19 | 1.08 | 2.02 | -1.98 | -1.33 | 2.04 | 3.62 | 5.43 | 9.18 | 12.88 | 14.65 | 16.18 | 19.40 | 20.01 |
| 38 | 29.64 | 0.19 | 1.15 | 2.01 | -1.83 | -1.15 | 2.36 | 3.99 | 5.84 | 9.64 | 13.36 | 15.12 | 16.65 | 19.84 | 20.45 |
| 39 | 30.11 | 0.19 | 1.24 | 2.00 | -1.71 | -0.99 | 2.68 | 4.36 | 6.26 | 10.11 | 13.84 | 15.60 | 17.12 | 20.29 | 20.89 |
| 40 | 30.58 | 0.19 | 1.33 | 1.98 | -1.64 | -0.87 | 2.99 | 4.74 | 6.68 | 10.58 | 14.32 | 16.07 | 17.59 | 20.73 | 21.32 |
